# Allergic Rhinitis and Its Relationship with IL-10, IL-17, TGF-*β*, IFN-*γ*, IL 22, and IL-35

**DOI:** 10.1155/2018/9131432

**Published:** 2018-03-06

**Authors:** P. Bayrak Degirmenci, S. Aksun, Z. Altin, F. Bilgir, I. B. Arslan, H. Colak, B. Ural, D. Solakoglu Kahraman, G. Diniz, B. Ozdemir, C. Kırmaz

**Affiliations:** ^1^Allergy Immunology Department, Tepecik Training and Research Hospital, Health Sciences University, Izmir, Turkey; ^2^Department of Biochemistry, Katip Celebi University, Izmir, Turkey; ^3^Internal Medicine Department, Tepecik Training and Research Hospital, Health Sciences University, Izmir, Turkey; ^4^Allergy Immunology Department, Ataturk Training and Research Hospital, Katip Celebi University, Izmir, Turkey; ^5^Ear, Nose, and Throat Department, Tepecik Training and Research Hospital, Health Sciences University, Izmir, Turkey; ^6^Nephrology Department, Tepecik Training and Research Hospital, Health Sciences University, Izmir, Turkey; ^7^General Surgery Department, Tepecik Training and Research Hospital, Health Sciences University, Izmir, Turkey; ^8^Pathology Department, Tepecik Training and Research Hospital, Health Sciences University, Izmir, Turkey; ^9^Allergy Immunology Department, Manisa State Hospital, Manisa, Turkey; ^10^Department of Internal Medicine, Division of Allergy Immunology, Manisa Celal Bayar University, Manisa, Turkey

## Abstract

**Background:**

We aimed in our study to research the role of new cytokines such as IL-35, IL-22, and IL-17 that may form a target for novel treatment approaches.

**Methods:**

IL-10, IL-17, TGF-*β*, IFN-*γ*, IL-22, and IL-35 serum levels of allergic rhinitis (AR) patients were measured using ELISA method. Allergic sensitization was demonstrated by the skin prick test. Patients only with olive tree sensitivity were evaluated for seasonal AR (SAR). Patients only with mite sensitivity were included in the study for perennial AR (PAR). AR clinic severity was demonstrated by the nasal symptom scores (NSS).

**Results:**

In total, 65 AR patients (patient group), having 31 PAR and 34 SAR patients, and 31 healthy individuals (control group) participated in the study. Cytokine levels between the patient group and the control group were compared; IL-17 (*p* = 0.038), IL-22 (*p* = 0.001), and TGF-*β* (*p* = 0.031) were detected as high in the patient group, and IFN-*γ* (*p* < 0.001) was detected as low in the patient group. When correlation analysis was made between age, gender, prick test result, NSS, AR duration, and cytokine levels in the patient group, a negative correlation was detected only between IFN-*γ* (*p* = 0.032/*r* = −0.266) level and NSS.

**Conclusions:**

Accompanied by the literature information, these results made us think that T cell subgroups and cytokines have an important role in AR immunopathogenesis. It is thought that future studies to be conducted relating to this subject will form new targets in treatment.

## 1. Introduction

Allergic rhinitis (AR) is an IgE-mediated chronic inflammatory disease of the upper airways characterized by symptoms of sneezing, rhinorrhea, nasal itching, and nasal obstruction. The development of allergic rhinitis requires an interaction between the environment, immune system, and genetic susceptibility. Several cells, cytokines, and chemokines orchestrate and maintain allergic inflammation. Cytokines play an essential role in mediating allergic inflammation. The importance of the T helper cell (Th) 2 cytokines in both the development of allergic sensitization and pathology of allergic inflammation is well established [1]. While healthy subjects are predominated by Th1-type cells, nasal mucosa and epithelial tissues of AR subjects are dominated by Th2-type lymphocytes [2].

Characteristic cytokine as known for Th1 cell IFN-*γ* is a key cytokine in bridging the innate and the adaptive arms of the immune system. In addition to its role in the development of a Th1-type response, IFN-*γ* plays a role in the regulation of local leukocyte-endothelial interactions [3].

Another Th subgroup of regulatory T cells (Tregs) has restrictive influences on both Th1 and Th2 cell-mediated inflammation. The lack of Tregs causes the emergence of allergic inflammation along with the increase in Th2 cells [4]. The Tregs bring the allergic inflammation under control by synthesizing IL-10 and the transforming growth factor-*β* (TGF-*β*) [5]. Allergic patients show an allergen-specific functional defect in Treg that promotes Th2 polarization and consequently IgE synthesis [6].

More recently, there is new evidence on the roles of Th17 cells, which are a distinct subpopulation of CD4+ T cells that produce IL-17A, IL-17F, IL-22, TNF-*α*, and IL-21 [7]. Studies seem to suggest that Th17 cells may be involved in the process of neutrophilic infiltration that occurs during the acute phase of allergic reaction. IL-17 was found to contribute to the induction of allergen-specific Th2 cell activation, eosinophil accumulation, and serum IgE production thus suggesting a regulatory role of IL-17A on the established Th2-driven allergic immune response [8]. However, the findings on IL-17 are ambiguous and the role of TH17-cells in AR remains unclear [9].

Lately, IL-22 has been shown to be specifically produced by Th17 cells. IL-22 exhibits both proinflammatory and anti-inflammatory properties depending on the environmental context [10]. While a number of studies have shown that IL-22 plays protective roles in host defense against infectious diseases, some studies have shown that IL-22 is involved in the development of autoimmune diseases [11]. To date, few reports have investigated the role of IL-22 in allergic diseases [12, 13].

IL-35 is a recently described cytokine and is a member of the IL-12 family. IL-35 is produced mainly by Tregs [14]. IL-35 is a novel anti-inflammatory cytokine suppressing the immune response through the proliferation of Tregs and suppression of Th17 cell function [15, 16].

The aforementioned cytokines are effective molecules in the shaping of allergic immune response. The pathogenic mechanisms involved in allergic sensitization are complex and still partially understood. There is no detected study in the literature that examines IL-10, IL-17, TGF-*β*, IFN-*γ*, IL-22, and IL-35 cytokines in patients with AR compared with a normal population and shows a broader connection with T lymphocyte subgroups. We aimed in our study to research the role of new cytokines such as IL-35, IL-22, and IL-17 that may form a target for novel treatment approaches.

There are studies in the literature evaluating allergic rhinitis cytokines in nasal lavage. However, due to technical insufficiency, we preferred to evaluate serum cytokine levels in this study [17].

## 2. Methods

This is a prospective study. Approval was received on May 5, 2013 from the Ethical Board for Clinical Research, Faculty of Medicine, Celal Bayar University, Turkey, and the Decision Reference Number 2013/153 was assigned. Written informed consent of all patients was obtained.

Diagnosis of AR was based on history, physical examination, and laboratory findings. The patients having applied for the adult allergy-immunology clinic in Tepecik Training Research Hospital and after medical history and physical examination, who were predicted to have AR, were administered skin prick test.

Allergic sensitization was demonstrated by the skin prick test. Skin prick tests were performed according to the EAACI guidelines for the most common inhalant allergens in Turkey, including house dust mites (*Dermatophagoides pteronyssinus* and *Dermatophagoides farinae*), fungi (*Aspergillus fumigatus*, *Alternaria alternata*, *Cladosporium herbarum*, and *Penicillium notatum*), grasses (*Lolium perenne*, *Fectuca pratensis*, *Phleum pratense*, *Poa pratensis*, and *Dactylis glomerata*), weeds (*Plantago lanceolata*, *Artemisia vulgaris*, *Rumex acetosa*, *Taraxacum vulgare*, and *Parietaria offıcinalis*), and trees (*Sambucus nigra*, *Populus alba*, *Ulmus scabra*, *Salix caprea*, *Fagus sylvatica*, *Carpinus betulus*, *Quercus robur*, *Fraxinus excelsior*, and *Olea europaea*) with histamine and diluent control (Allergopharma Ltd, Reinbek, Germany) [18].

Allergic rhinitis clinic severity was demonstrated by the nasal symptom scores (NSS). NSS (sneezing, nasal obstruction, nasal itching, and watery nasal discharge; graded as 0 = none, 1 = mild, 2 = moderate, and 3 = severe, up to a maximum of 12 points) were recorded for all patients during the pollen season, when their symptoms were at their worst.

In skin prick test, out of the patients detected to have sensitivity for only mite and olive tree pollen, those having symptoms for 2 years were included in the study. Patients known to have had any immunosuppressive treatment within the last 1 month, to have had antihistamine treatment within the last 10 days, to have smoked, to have any active infection, and to have another chronic disease such as chronic rhinosinusitis, nasal polyposis, and cancer were excluded from the study.

AR patients were divided into two groups according to allergen sensitivities as seasonal allergic rhinitis (SAR) and perennial allergic rhinitis (PAR). Patients only with olive tree sensitivity and having seasonal symptoms were evaluated for SAR. Patients only with mite sensitivity and having year-long symptoms were included in the study for PAR.

Peripheral blood samples were taken for evaluation of serum cytokine level in the period with the highest symptom scores for both patient groups, and cytokine measurement was performed with ELISA method.

The control group consisted of volunteer people with similar age and gender range with patients, with no allergic and systemic disease history and with no drug use within the last one month. Skin prick tests of the control group were negative.

### 2.1. Detection of Cytokines

The plasma IL-10, IL-17, TGF-*β*, IFN-*γ*, IL-22, and IL-35 levels were measured by ELISA (Beckman Coulter DXI-800, USA) according to the manufacturer's protocols (IL-10, IL-35, TGF-*β*, and IFN-*γ*, ELISA kits, BioSource, Nivelles, Belgium; IL-22 ELISA kit, Bender MedSystems, Vienna, Austria, Europe; and IL-17 ELISA kit, RayBio, Ray Biotech Inc., GA, USA). The minimal detectable concentrations were 2 pg/ml for IL-35, 1 pg/ml for IL-10, 10 pg/ml for IL-22, 15.6 pg/ml for TGF-*β*, 10 pg/ml for IL-17, and 4 pg/ml for IFN-*γ*. All samples were detected in duplicate.

## 3. Statistics

In the analysis of the data, SPSS22.0 (IBM Corporation, Armonk, New York, United States) program was used. Shapiro-Wilk test was used for conformity of data to normal distribution and Levene's test for variance homogeneity, and parametric methods were used in analysis of variables having homogenous variance and normal distribution, and nonparametric methods were used in analysis of variables having nonhomogeneous variance and abnormal distribution. In comparison of two independent groups, independent-samples *t*-test was used with Bootstrap results while Mann–Whitney *U* test was used with Monte Carlo simulation technique. Pearson's correlation test was used to examine the correlation of variables with each other. In the comparison of categorical data, Fisher's exact test was tested with Monte Carlo simulation technique. In order to determine the cause-and-effect relationship with diatom descriptive variables of categorical response variable, logistic regression test was used with Bootstrap results. Quantitative data were presented in the tables as mean ± SEM (standard deviation) and median range (maximum–minimum) values. Categorical data were expressed as *n* (number) and percentages (%). The data were examined in 95% reliability range and *p* value is accepted as significant if below 0.05.

## 4. Results

Patients were included in the study between the dates of March 2014 and June 2015. In total, 65 AR patients (patient group), having 31 PAR and 34 SAR patients, and 31 healthy individuals (control group) participated in the study. Sociodemographical data are shown in Table 1.

**Figure 1:**
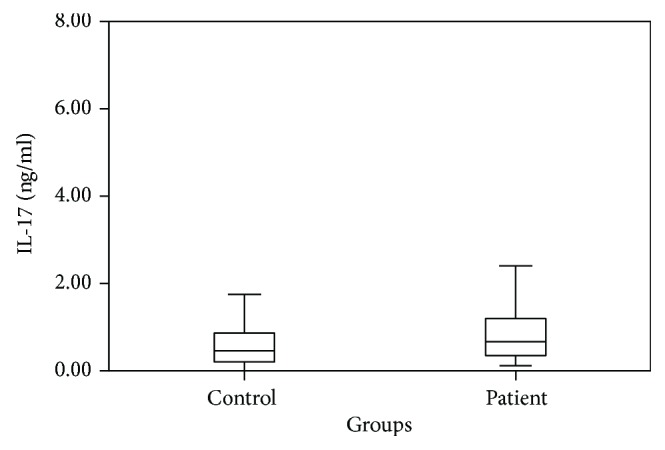
Patient and control group comparison of IL-17 levels.

**Figure 2:**
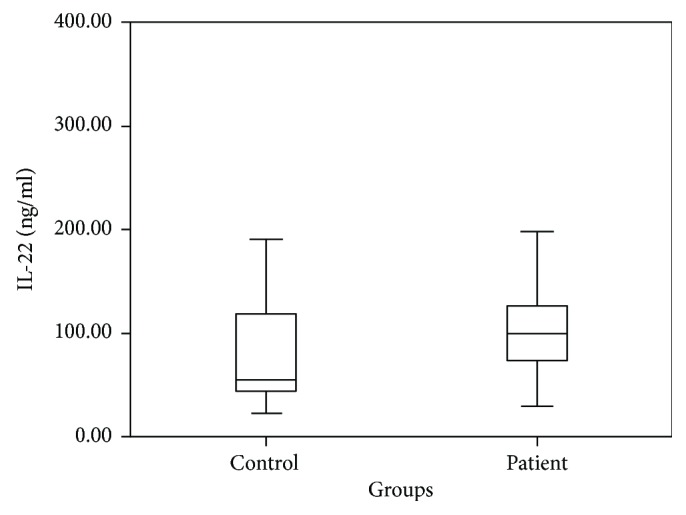
Patient and control group comparison of IL-22 levels.

**Figure 3:**
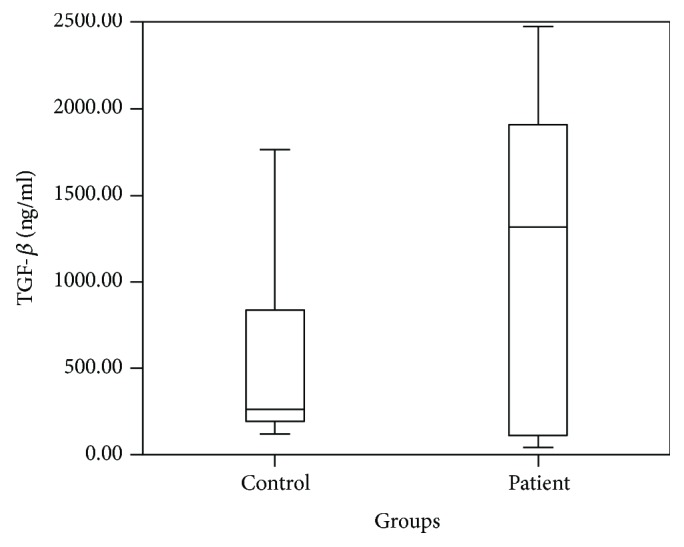
Patient and control group comparison of TGF-*β* levels.

**Figure 4:**
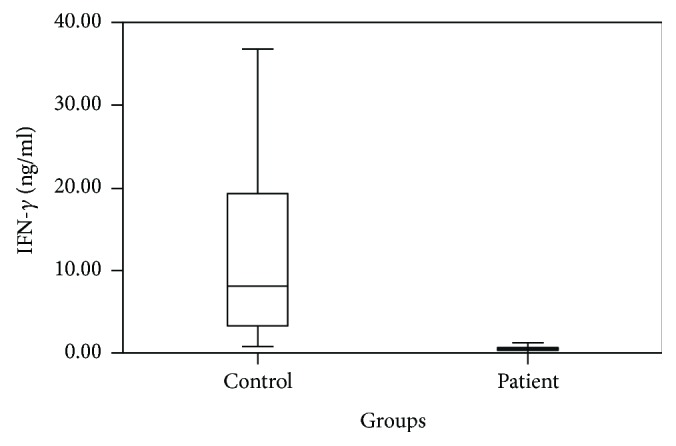
Patient and control group comparison of IFN-*γ* levels.

Cytokine levels between the patient group and the control group were compared using independent *t*-test; IL-17 (*p* = 0.038), IL-22 (*p* = 0.001), and TGF-*β* (*p* = 0.031) were detected as high in the patient group and IFN-*γ* (*p* < 0.001) was detected as low in the patient group. Cytokine levels of the AR patients and the control group are shown in Table 2. Patient-control group comparison of IL-17, IL-22, TGF-*β*, and IFN-*γ* levels is shown in Figures 1, 2, 3, and 4.

When correlation analysis was made between age, gender, prick test result, NSS, AR duration, and cytokine levels in the patient group, a negative correlation was detected only between IFN-*γ* (*p* = 0.032/*r* = −0.266) level and symptom score. It is shown in Table 3.

According to prick test results, the patient group was divided into two groups as PAR and SAR. In the comparison of the cytokine levels of PAR and SAR patients, no statistically significant difference was detected.

## 5. Discussion

There are several studies in the literature describing the relationship between AR and cytokines. It is known that AR is a Th2-dominant disease and there are many studies exhibiting the relationship of cytokines with AR such as Th2-based IL-4 and IL-13. We desired in this study to evaluate different Th subgroups and cytokines in AR patients.

At the end of our study, IL-17(*p* = 0.038), IL-22 (*p* = 0.001), and TGF-*β* (*p* = 0.031) were detected as high in the patient group and IFN-*γ* (*p* < 0.001) was detected as low in the patient group. Moreover, a negative correlation between IFN-*γ* (*p* = 0.032/*r* = −0.266) level and NSS was observed.

As in majority of literature studies, IFN-*γ* was used to measure Th1-mediated immune response in AR patients. Similar to literature information, serum IFN-*γ* level was detected lower in AR patients that in the control group [19]. This result makes us think that there is downregulation in Th1-mediated immune response in periods when symptoms of AR patients are severe.

Tregs responsible for enabling immune homoeostasis with immune regulatory mechanisms suppress cytokines having a role in Th1- and Th2-mediated responses and allergic immune responses [20]. In our study, IL-10 and TGF-*β* were used to determine Treg functions. TGF-*β* levels of patients evaluated in the pollen season in our study was detected as higher than those of controls; however, no significant difference was detected between IL-10 levels and the related patient and control group. In the study of Ciprandi and colleagues, *Parietaria*- and grass-sensitized AR patients were evaluated in 2 groups, namely, in season and out of season. Serum TGF-*β* level of AR patients out of the pollen season was detected as significantly low while it was shown that TGF-*β* level increased in the pollen season [21]. In another study, serum IL-10 and TGF-*β* levels of AR patients were detected as lowed than those of controls; also, a negative correlation was detected between SS and IL-10 and TGF-*β* levels, and a positive correlation between SS and IL-17 [22].

TGF-*β* has multiple regulatory effects on T cell proliferation, antigen presentation, and expression of costimulatory proteins and is relevant for the function of T regulatory cells.

In the literature, successful subcutaneous and sublingual immunotherapy is followed by increased production of both IL-10 and TGF-*β* [23].

IL-17 and IL-22 were used in our study to evaluate Th17 mediating the arising of neutrophilic inflammation. IL-17 and IL-22 serum levels were detected as higher in AR patients than controls in our study. In many studies of literature, serum IL-17 and IL-22 levels of AR patients are higher than those of control groups which is similar to our study [24–27]. In the study of Ciprandi and colleagues, serum IL-17 levels of AR patients were detected higher than those of controls and also, a positive correlation was indicated between serum IL-17 level and SS [28]. In another study, it was shown that serum IL-17 level in AR patients was higher while TGF-*β* level was lower than that in controls [29]. In our study and in many studies of literature mentioned above, it was shown that Th17 cells were active in a period when both SAR and PAR patients are symptomatic. This result arises the idea that Th17 cells and Th17-derived cytokines were important in AR immunopathogenesis and it should be evaluated with advanced studies.

In our study, IFN-*γ* level was detected lower in AR patients and also, a negative correlation was detected between IFN-*γ* level and SS. These results made us think that symptoms of patients worsened as Th1 activity decreased in AR patients. However, this information is different from literature information. In many studies of literature, a positive correlation was detected between serum IL-17 level and SS and a negative correlation was detected between IL-10 and TGF-*β* levels [23, 28].

It was shown that a recently identified immunosuppressive cytokine, IL-35, has an important role in diseases such as experimental colitis and collagen-induced arthritis [30, 31]. More recent findings have suggested that IL-35 may also play a central role in asthma, as it effectively attenuated airway inflammation and IgE production induced by an allergen-specific memory/effector Th2 cell line [32]. Furthermore, IL-35 production by inducible costimulator-positive Treg has been shown to suppress IL-17 production and reverse established IL-17-dependent airway hyperreactivity in asthma [33]. However, the role of IL-35 in AR immunopathogenesis is still indefinite. There are murine model studies conducted relating to AR and IL-35. In these studies, it was stated that IL-35 directly or indirectly suppressed Th2-type immune response or allergic inflammation and it may be a target for novel treatment models [34, 35]. Considering the limited number of human studies, Wang and colleagues showed in their study that IL-35 suppressed IL-4 secretion in allergic asthma patients and IL-35 level was lower than that in healthy controls [36]. In our study, despite being not statistically significant, IL-35 serum level was detected lower in AR patients than controls.

As a result of our study, no significant difference was detected between the SAR and PAR patient groups in terms of cytokine levels. In both patient groups evaluated in the symptomatic period, detection of IL-17, IL-22, and TGF-*β* as high arises the idea of the activation of Th17 and Treg and detection of IFN-*γ* as low arises the idea of Th1 inhibition. IL-35 level, despite being not statistically significant, was detected as lower in the patient group than controls. Low detection of IL-35, which was shown to suppress Th2-mediated allergic airway inflammation and IL-17-mediated immune response, in AR patients makes us think that IL-35 secretion deficiency has a role in AR pathogenesis. It is obvious that T cell subgroups and cytokines have an important role in AR immunopathogenesis.

The mainstay of current treatment strategies of AR includes allergen avoidance, pharmacotherapy, and allergen-specific immunotherapy as defined by AR and its impact on asthma guidelines [37].

It is thought that future studies to be conducted relating to this subject will form new targets in treatment.

## Figures and Tables

**Table 1:** Demographic of the groups.

Variables	Control (*n* = 31)	Patient (*n* = 65)
Sex, F/M (female/male)	16 (51.6%)/15 (48.4%)	36 (55.4%)/29 (44.6%)
Age (mean ± SEM)	31.16 ± 7.95	31.92 ± 9.64
Duration of AR		6.78 ± 2.53
Symptom score (mean ± SEM) (min–max)		9.69 ± 1.93 (6–12)
nPAR/nSAR		31 (47.7%)/34 (52.3%)

**Table 2:** Cytokine levels of AR patients and control group.

Cytokines (ng/ml)	Control (*n* = 31)	Patient (*n* = 65)	*p* value
IL-17	0.45 (1.75–0.001)	0.68 (7.18–0.11)	*0.038*
IL-35	59.9 (258–33.53)	57.71 (110.81–22.87)	0.104
IL-22	55.6 (351.14–22.9)	99.3 (198.26–29.9)	*0.001*
TGF-*β*	262.43 (2013–122.52)	1315 (2476.3–45.3)	*0.031*
IL-10	4.9 (9.5–1.4)	5.11 (24.27–1.64)	0.888
IFN-*γ*	8.13 (36.71–0.89)	0.5 (2.6–0.044)	*<0.001*

**Table 3:** Correlation analysis between cytokine levels, age, disease duration, and symptom score of AR patients.

Cytokines	Age		AR duration		Symptom score	
	*r*	*p*	*r*	*p*	*r*	*p*
IL-17	−0.151	0.229	−0.009	0.944	0.192	0.125
IL-35	−0.183	0.145	0.078	0.537	−0.003	0.983
IL-22	0.180	0.151	−0.024	0.851	0.048	0.702
TGF-*β*	0.157	0.212	−0.043	0.733	0.166	0.186
IL-10	−0.145	0.249	−0.198	0.113	−0.032	0.799
IFN-*γ*	−0.221	0.076	−0.175	0.164	*−0.266*	*0.032*

Pearson's correlation test *r*: correlation coefficient.
